# DNA vaccine based on conserved HA-peptides induces strong immune response and rapidly clears influenza virus infection from vaccinated pigs

**DOI:** 10.1371/journal.pone.0222201

**Published:** 2019-09-25

**Authors:** Marta Sisteré-Oró, Sergi López-Serrano, Veljko Veljkovic, Sonia Pina-Pedrero, Júlia Vergara-Alert, Lorena Córdoba, Mónica Pérez-Maillo, Patrícia Pleguezuelos, Enric Vidal, Joaquim Segalés, Jens Nielsen, Anders Fomsgaard, Ayub Darji

**Affiliations:** 1 IRTA, Centre de Recerca en Sanitat Animal (CReSA, IRTA-UAB), Campus de la Universitat Autònoma de Barcelona, Bellaterra, Barcelona, Spain; 2 Biomed Protection, Galveston, Texas, United States of America; 3 UAB, Centre de Recerca en Sanitat Animal (CReSA, IRTA-UAB), Campus de la Universitat Autònoma de Barcelona, Bellaterra, Barcelona, Spain; 4 Departament de Sanitat i Anatomia Animals, Facultat de Veterinària, UAB, Bellaterra (Cerdanyola del Vallès), Barcelona, Spain; 5 Virus Research and Development Laboratory, Department of Virus and Microbiological Special Diagnostics, Statens Serum Institut, Artillerivej, Copenhagen S, Denmark; Icahn School of Medicine at Mount Sinai, UNITED STATES

## Abstract

Swine influenza virus (SIVs) infections cause a significant economic impact to the pork industry. Moreover, pigs may act as mixing vessel favoring genome reassortment of diverse influenza viruses. Such an example is the pandemic H1N1 (pH1N1) virus that appeared in 2009, harboring a combination of gene segments from avian, pig and human lineages, which rapidly reached pandemic proportions. In order to confront and prevent these possible emergences as well as antigenic drift phenomena, vaccination remains of vital importance. The present work aimed to evaluate a new DNA influenza vaccine based on distinct conserved HA-peptides fused with flagellin and applied together with Diluvac Forte as adjuvant using a needle-free device (IntraDermal Application of Liquids, IDAL®). Two experimental pig studies were performed to test DNA-vaccine efficacy against SIVs in pigs. In the first experiment, SIV-seronegative pigs were vaccinated with VC4-flagellin DNA and intranasally challenged with a pH1N1. In the second study, VC4-flagellin DNA vaccine was employed in SIV-seropositive animals and challenged intranasally with an H3N2 SIV-isolate. Both experiments demonstrated a reduction in the viral shedding after challenge, suggesting vaccine efficacy against both the H1 and H3 influenza virus subtypes. In addition, the results proved that maternally derived antibodies (MDA) did not constitute an obstacle to the vaccine approach used. Moreover, elevated titers in antibodies both against H1 and H3 proteins in serum and in bronchoalveolar lavage fluids (BALFs) was detected in the vaccinated animals along with a markedly increased mucosal IgA response. Additionally, vaccinated animals developed stronger neutralizing antibodies in BALFs and higher inhibiting hemagglutination titers in sera against both the pH1N1 and H3N2 influenza viruses compared to unvaccinated, challenged-pigs. It is proposed that the described DNA-vaccine formulation could potentially be used as a multivalent vaccine against SIV infections.

## Introduction

Swine influenza viruses (SIVs) are single-stranded, negative sense segmented RNA viruses that belong to the *Influenzavirus A* genus within the *Orthomyxoviridae* family. SIVs are common throughout pig populations worldwide and they generally cause coughing, sneezing, nasal discharges, fever, conjunctivitis, respiratory difficulties, lethargy, decreased food intake [1–4] and, in some instances, abortions in pregnant sows due to fever [5,6].

Even though pigs recover rapidly from clinical signs caused by SIVs, influenza has been recognized as an important cause of economic loss for the pig industry. The economic consequences are attributed to morbidity rates up to 100% [1] linked to the retarded animal growth and a prolonged finishing period [7]. Besides economic impact, the rapid genetic evolution of these viruses makes their control even more mandatory. On one hand, the antigenic drift phenomena allow the acquisition of point mutations in the hemagglutinin (HA) gene and, to a lesser extent, in the neuraminidase (NA) gene. These mutations may generate mutants able to escape the vaccine-induced immunity [5,8,9]. On the other hand, pigs are considered as “mixing vessels” due to the presence and distribution of both α2,3- and α2,6- sialic acid receptors in their respiratory tract [10,11], which might lead to the emergence of new assortment of viruses, like the influenza virus A H1N1 (pH1N1) that emerged in 2009 [12–14].

Vaccination is the most effective approach employed to control SIV infections. Currently, SIV vaccines available on the market are represented by conventional inactivated-type vaccines encompassing strains of H1N1, H1N2 and/or H3N2 subtypes, the most prevalent subtypes in swine herds [15–18]. In spite of the reduction in clinical signs and high antibody titers induced both in serum and alveolus, commercial vaccines have some weaknesses [19,20]. Apart from not being sufficiently protective when the strain does not closely match with the ones included in the vaccine product, they do not confer protection when facing against heterovariant or heterosubtypic challenges [21–24]. Some research studies have hypothesized that this matter could be related to the lack of cell-mediated and/or mucosal responses provided by the inactivated-type vaccines [23,25,26]. Moreover, it is also evidenced that maternally derived antibodies (MDA) may interfere in the development of immunity provided by vaccination [27,28]. Fundamentally, piglets with MDA at vaccination showed prolonged flu-like clinical signs, more severe SIV-pneumonia and suppression of both humoral and cellular responses in comparison to vaccinated MDA-seronegative piglets [27].

For these reasons, many efforts have been directed to design a universal vaccine that should cover all relevant subtypes of influenza, including varying field strains, and able to avoid the likelihood of emergence of forthcoming pandemic strains. The ideal vaccine should also overcome MDA interference. Currently, conserved areas of the virus proteins are targeted for the design of such vaccine [29–33]. In fact, those designs are based on combining different well-conserved epitopes to improve their protection and strain coverage.

Our group has defined new vaccine strategies utilizing conserved epitopes of the influenza A virus (IAV), specifically from the HA protein [29]. In the present work, with the aim to improve our vaccine prototype, a DNA vaccine encoding a combination of HA-conserved immunogenic epitopes along with flagellin (VC4-flagellin) was designed. Selection of these HA peptide epitopes (from H7, H5N1 or pH1N1) was based on the encoded informational spectrum frequencies that are common for the IVs judged by informational spectrum methodology (ISM). Previously, it has been shown that antigens which share a common frequency component in their informational spectra are immunologically cross-reactive [34].

Instead of immunizing animals with this new construct via intramuscular, a needle-free approach (IntraDermal Application of Liquids, IDAL®) was used. This administration route is safer because of the needle-free system and easy to be used in large-scale vaccination programs [35,36]. Moreover, to test the broad-based immunity and the protective efficacy of the vaccine, both MDA-seropositive and MDA-seronegative animals were used and were challenged with either pH1N1 or SwH3N2 to assess the cross-protective effect of the vaccine in two different experiments.

Experiment I was developed under a more favorable scenario: SIV-seronegative pigs challenged with a homologous virus (pH1N1) for the HA-peptide: SLPFQNIHPITIGKCPKYVKSTKLRLATGLRNV. By contrast, experiment II was designed to evaluate vaccine efficacy when the MDA were present and a heterologous virus was challenged (SwH3N2), representing a more unfavorable scenario. Subtypes H1 and H3 were inoculated since they are most common subtypes circulating in swine herds.

Herewith, we demonstrated that vaccination with VC4-flagellin DNA induced high titers of seroprotective/neutralizing antibodies and contributed in reducing the viral shedding of the vaccinated pigs in presence and absence of MDA.

## Materials and methods

### Immunogen and expression vector construction

Four conserved HA-peptides were predicted *in silico* by the Informational Spectrum Methodology (ISM) [37,38] and expressed along with a flagellin-derived construct, which was also designed by the ISM bioinformatics platform [39]. The predicted peptides along with the flagellin were organized in tandem to construct the multipeptide: PQRERRRKKRGLFGAIAGVEVVNATETVERTNIPRICSKGKRTVDLGQCGLLTIQVGANDGETIDIDLKQINSQTLSSSGSSGSSGSSIDAALAQVDALRSDLGAVQNRFNSAGVEVVNATETVERTNIPRICSKGKRTVDLGQCGLSLPFQNIHPITIGKCPKYVKSTKLRLATGLRNV, designated hereafter as VC-4-flagellin. This sequence was reverse-translated with codon optimization for swine expression and cloned into the pCDNA3.1(+) plasmid (GenScript, NJ, USA). Table 1 describes each of the four predicted HA epitopes. Expression of the construct was controlled by in vitro transfection and purification of the plasmid at large-scale production was performed with the EndoFree plasmid Gigakit (Qiagen, Barcelona, Spain). Purified plasmid DNA was quantified by using Biodrop μLITE Spectrophotometer (BioDrop Ltd, Cambridge, UK), resuspended in sterile saline solution and kept at -20°C until used.

**Table 1:** Amino acid sequences from the HA-epitopes used in the VC4-flagellin construct; aa positions from their respective consensus IV are also indicated.

HA-epitopes	Aa positions*	Consensus virus subtype	GenBank Id
PQRERRRKKRGLFGAIA	337–357	H5N1	AAC32098.1
GVEVVNATETVERTNIPRICSKGKRTVDLGQCGLLTI	41–77	H7N1	AGT40751.1
	37–71	H7N7	ACN80240.1
	33-67/37-71	H7N8	AFP99768.1
	41–75	H7N9	ASV61404.1
GVEVVNATETVERTNIPRICSKGKRTVDLGQCGL	41–74	H7N3	APD70004.1
		H7N6	ANK78016.1
		H7N7	ANC28237.1
		H7N9	AJU15322.1
SLPFQNIHPITIGKCPKYVKSTKLRLATGLRNV	168–200	H1N1	ALN12227.1

*the most common aa positions of HA-epitopes; though they also could be encountered in other aa positions.

The aa positions shown are according to the reference cited from the GenBank database. Abbreviations: aa = amino acid; HA = hemagglutinin; Id = identification

### Cells and antigens

Madin-Darby Canine Kidney (MDCK, ATCC CCL-34) cells were used to prepare viral stocks and to perform the seroneutralization assays on BALFs. MDCKs were grown in Dulbecco’s Modified Eagle Medium (DMEM) supplemented with 10% foetal bovine serum (FBS), 1% penicillin/streptomycin and 1% L-glutamine.

Hemagglutinins of A/California/04/09(H1N1)pdm09 and A/Aichi/2/1968 (H3N2) were acquired from SinoBiological (Cat no. 40340-V08B and 11707-V08H; respectively, SinoBiological Inc., PA, USA) and were used as purified antigens for in-house enzyme-linked immunosorbent assay (ELISA) test developments.

### Ethics statement

Experiments with SIVs were performed at the Biosafety Level 3 (BSL-3) facilities at IRTA-CReSA (Barcelona, Spain). The experiment protocols were supervised and approved by the Ethical and Animal Welfare Committee of Institut de Recerca i Tecnologia Agroalimentàries (IRTA) and the Ethical Commission of Animal Experimentation of the Autonomous Government of Catalonia. In addition, both conducted research studies followed the Directive UE 63/2010, the Spanish Legislation, RD 53/2013, the Catalan Law 5/1995, Decree 214/1997 and the ARRIVE guidelines checklist (S1 Checklist).

Animals from Experiment II were housed in a conventional farm during the immunization, and transferred to BSL-3 facilities one week prior to challenge (adaptation period).

In both of the experiments, animals were fed with food and water *ad libitum* and were not treated with anesthetics or analgesics since they were not suffering from the disease and/or experimental manipulation.

### Animal experimental design

Two experiments were carried out to assess the DNA-vaccine efficacy *in vivo* (Table 2). Clinically healthy pigs purchased from commercial farms were selected and tested for presence of specific antibodies in sera against the influenza nucleoprotein (NP) using the ID Screen ® Influenza A Antibody Competition ELISA (ID VET, France) kit. SIV-seronegative animals were selected for Experiment I. For MDA positive pig studies, piglets were obtained from vaccinated sows and were controlled for having NP antibodies by ELISA. All pigs had antibody titers against H1 and H3 subtypes with an average OD 450 nm of 0.6–0.8 without bias towards maternal antibody titers. Moreover, in both cases, RT-qPCR (see section 2.8.) was also determined to ensure animals were not exposed to IV.

**Table 2:** Schematic outline of Experiments I and II.

Experiment	Groups	N° animals (n)	MDA	Challenged virus
Experiment I	Group A: unvaccinated	5	Absence	pH1N1
	Group B: pCDNA3.1(+)-VC4-flagellin	5		
Experiment II	Group A: unvaccinated	6	Presence	SwH3N2
	Group B: pCDNA3.1(+)-VC4-flagellin	6		

Abbreviations: MDA = maternally derived antibodies

#### Experiment I (SIV-seronegative pigs/challenged with pH1N1)

Ten 5-to-6-week-old male pigs seronegative against SIV were randomly divided into two groups: animals 1–5 (Group A, n = 5) and animals 6–10 (Group B, n = 5) and were housed together in the same box. Animals from group B were immunized twice with a 21-day interval period. The immunizations consisted of 600 μg (3 IDAL® shots/200 μg/100 μL animal) of the VC4-flagellin DNA construct mixed in a ratio of 1:1 (v/v) with Diluvac Forte® adjuvant (MSD Animal Health, Salamanca, Spain) applied with the IDAL® device (MSD Animal Health) on the dorsal side of the back of each pig [36]. Animals from A group were sham-vaccinated by administration of 2 mL/animal of PBS+DiluvacForte®. Two weeks after booster immunization, all pigs were intranasally challenged with pH1N1. All animals were euthanized seven days post-inoculation (dpi) with an overdose of pentobarbital followed by exsanguination.

#### Experiment II (SIV-seropositive pigs/challenged with SwH3N2)

Twelve 4-week-old SIV-seropositive male or female pigs were separated into two groups: animals 1–6 (Group A, n = 6) and animals 7–12 (Group B, n = 6). Animals from groups A (sham-vaccinated) and B (VC4-flagellin DNA-vaccinated) were immunized as described in Experiment I. Two weeks after the second immunization, animals were intranasally challenged with SwH3N2. Upon transferred to BSL3 installations, both groups were accommodated together. Animals were euthanized with an overdose of pentobarbital followed by exsanguination either at 7 or 14 dpi.

### Sampling and clinical records

Flu-like clinical signs were monitored during all the experiment. Fever was considered when rectal temperatures reached values above 40°C [40]. The sampling schedule for both experiments is represented in Table 3. Briefly, nasal swabs were collected to determine the presence of viral RNA and sera were collected to analyse the humoral immune response at different time-points.

**Table 3:** Sampling schedule for experiments I and II.

Sample	Experiment	0 PVD	21 PVD	35 PVD	5 dpi	7 dpi	11 dpi	14 dpi
**Sera**	Experiment I	✓	✓	✓		✓		
	Experiment II	✓	✓	✓		✓		✓
**Nasal swabs**	Experiment I	✓		✓	✓	✓		
	Experiment II	✓		✓	✓	✓	✓	✓
**BALF**	Experiment I							
	Experiment II					✓		✓
**Lung tissues**	Experiment I					✓		
	Experiment II					✓		✓

Abbreviations: BALF = bronchoalveolar lavage fluid; dpi = days post-inoculation; PVD = post-vaccination days

Complete necropsies were performed at the indicated times after infection (7dpi, Experiment I; 7 or 14 dpi, Experiment II). Gross pictures were taken from both sides of the lung to assess the macroscopiclung lesion score. Subsequently, three lung samples were collected (apical, middle and diaphragmatic lobes) from the left lung and fixed by immersion in 10% neutral buffered formalin to perform histopathological analysis. BALFs were also collected immediately from the right lung after post-mortem examination [41]. The BALF supernatants obtained were stored at -80°C to investigate antibody response (IgG and IgA) and to assess seroneutralizing titres against the challenged virus.

### Pathological procedures

Only in experiment II, the macroscopically affected lung area (%) from each individual was quantified by image analysis (IA) (ImageJ ® online free software) as previously described [42]. Formalin fixed tissues from the animals of both of the experiments were dehydrated and embedded in paraffin wax, sectioned at 3–5 μm and stained with hematoxylin-eosin (HE) for examination under light microscopy. In all lung samples, a semi-quantitative scoring method was determined as previously described [43].

### SIVs and inoculum preparation

The viruses used for inoculation were the pH1N1 virus (A/Catalonia/63/2009 H1N1 IV) [GenBank GQ464405-GQ464411 and GQ168897] and the SwH3N2 (A/swine/Spain/003/2010 H3N2 IV) [GenBank JQ319724 and JQ319726]. The infectious virus titres were determined by following the Reed and Muench methodology [44]. All pigs were intranasally inoculated with a total dose of 10^6^TCID_50_/mL (diluted in 3 mL saline solution and delivering a final volume of 1.5 mL/nostril using a mucosal atomisation device (MAD® Nasal; Teleflex ® Inc. NC, USA) to mimic aerogenous infection.

### Quantitative real-time RT-PCR (RT-qPCR)

Viral RNA quantification was performed in nasal swab samples using the NucleoSpin RNA isolation kit (MACHEREY-NAGELGmbH&CoKG, Düren, Germany) following the manufacturer’s instructions. Subsequently, a TaqMan RT-qPCR designed to detect influenza viruses (IVs) using the PCR primers and hydrolysis probe already described [45] was run in a Fast7500 equipment (Applied Biosystems, CA, USA) with the conditions already set and described [14].

### IgGs and IgAs enzyme-linked immunosorbent assays (ELISAs)

To assess IgG antibody responses against the purified antigens from H1N1 and H3N2 in sera and BALFs samples, specific ELISA tests were developed. Briefly, 96 well plates were coated with 2 μg/mL of each HA antigen diluted in 50 mM sodium bicarbonate buffer and incubated overnight at 4°C. After blocking with 3%BSA/PBS (100μL/well) for 1 hour at room temperature (RT) either serum from individuals diluted at 1:100 or neat BALFs samples were added (50μl/well) to the coated plate, followed by 1 hour incubation at RT. Plates were washed three times with 1% Triton X-100/PBS, and anti-pig IgG (whole molecule)-Peroxidase (Sigma-Aldrich, MO, USA) diluted 1:10,000 was added to wells followed by 30 minutes incubation at 37°C. After washing the plates four times (1% Triton X-100/PBS), 50 μL of 3, 3’, 5, 5’-tetramethylbenzidine (TMB) substrate solution was added to the wells and allowed to develop protected from light exposure for 10 minutes. Reaction was stopped by adding 50 μL of 1 NH_2_SO_4_ and the optical density (OD) was measured at 450 nm. Each sample was run in triplicates.

An in-house ELISA to detect mucosal response (IgA) against hemagglutinins from H1 and H3 subtypes was run in BALF samples. For that purpose, a previous protocol was followed with few modifications and by means of using IgA antibody (Cat no. AA140p; AbDSerotec, Oxford, UK) [46]. Briefly, the high-binding 96-well plates (Costar, Corning Incorpororated, NY, USA) were coated with 2 μg/mL of each HA in 50 mM sodium bicarbonate buffer and incubated overnight at 4°C. Samples were diluted 1:1 with blocking buffer.

### Hemagglutination inhibition (HI) assay

HI titers were obtained following the standard protocol instructions out of the World Organization for Animal Health (2012) using chicken red blood cells and 4 hemagglutination units of either pH1N1 IV or SwH3N2 IV. All sera were analyzed in duplicates. Positive and negative reference sera (purchased at the GD Animal Health, Deventer, The Netherlands) were used to validate the technique. “Seroprotective” titer (HI≥40) has been used as a criteria of immunogenicity in a vaccine and standard for licensure [47–50].

### Serum neutralization test (SNT)

MDCK cells were seeded into 96- well tissue culture plates to achieve confluence the following day. After 24 hours, BALFs samples were inactivated at 56°C for 30 minutes and serially diluted two-fold up to 1: 2,560 dilution using DMEM supplemented with 1% penicillin/streptomycin and 1% L-glutamine. In parallel, to promote a proper cleavage of the hemagglutinin protein, the H3N2 virus was trypsinized using porcine-trypsin (Sigma-Aldrich, MO, USA) for 30 minutes at 37°C. After this step, the virus was added to the diluted BALFs to yield final concentrations of 100 TCID_50_/well. Serum-virus mixtures were incubated at 37°C temperature for 2 hours and were added to PBS 1X washed MDCK cells. Media controls (no virus) and virus controls (no serum) were included on each plate. Reference positive and negative sera against H3N2 (GD Animal Health, Deventer, The Netherlands) were also incorporated. Each sample dilution was plated in duplicates. After an incubation period of 7 days, the plates were read. SNT titers were calculated as 50% endpoints for the greatest serum dilution giving complete inhibition of the virus growth [44].

### Flow cytometry

In order to identify the phenotype of T cells, peripheral blood mononuclear cells (PBMCs) were isolated before the challenge from whole blood by density centrifugation using Histopaque®-1077 gradient (Sigma-Aldrich, MO, USA) and performed the flow cytometry using monoclonal antibodies (mAbs). Cell numbers were calculated using a dye solution and the cell concentration was adjusted to 10^6^ cells/well, and single- or double-stained with surface antibodies diluted in PBS 1% anti-CD4 (clone 74-12-4, IgG2b) Alexa Fluor® 647-labelled (BD Pharmingen^™^, CA, USA) and anti-CD8 (clone 76-2-11, IgG2a) fluorescein isothiocyanate (FITC)-labelled (BD Pharmingen^™^, CA, USA). Cells were acquired by means of FACSCalibur (Becton Dickinson FACSAria I) (Becton Dickinson, CA, USA), and the positive frequencies analyzed by FACSDiva software, version 8.01. Gated images of different cell populations are shown in S1 Fig.

### Statistical analyses

Mean and standard deviations of studied parameters were calculated with Excel 2007 (Microsoft Office). All data obtained were first normalized by the Shapiro-Wilk test and the t-test (in case of normally distributed data) or the Wilcoxon test (in case of non-normally distributed data)and were subsequently used to compare A and B groups within each experiment. Statistical analyses were performed using the R statistical software (http://cran.r-project.org/) and the significance was depicted depending on the significance threshold obtained: *P*<0.05 (*), *P*<0.01 (**), *P*<0.001 (***) and *P*<0.0001 (****).

## Results

### Experiment I (SIV-seronegative pigs/challenged with pH1N1):

#### Clinical and pathological evaluation

Previous to the challenge, all animals were clinically healthy. Upon challenge, one animal out of five (pig 1) from the unvaccinated group had fever at 6 dpi and also one animal (pig 8) from the VC4-flagellin vaccinated group had fever but only at 2 dpi. Also, one animal (pig 2) from the unvaccinated group displayed loose feces at 7 dpi. No other clinical signs were recorded.

Challenge with pH1N1 caused subclinical infection in all pigs and minor histopathological changes observed at the necropsy. No differences in the severity of microscopic lung lesions between vaccinated and unvaccinated animals were recorded. Apart from the lung scorings based on broncho-interstitial pneumonia, other pathological findings were documented. Multiple abscesses were visualized in animal 7; animal 8 had fibrous pleuritis and animal 10 showed pulmonary congestion and edema. All three animals belonged to the VC4-flagellin vaccinated group.

#### Vaccination using VC4-flagellin limited or reduced pH1N1 viral load

**Fig 1:**
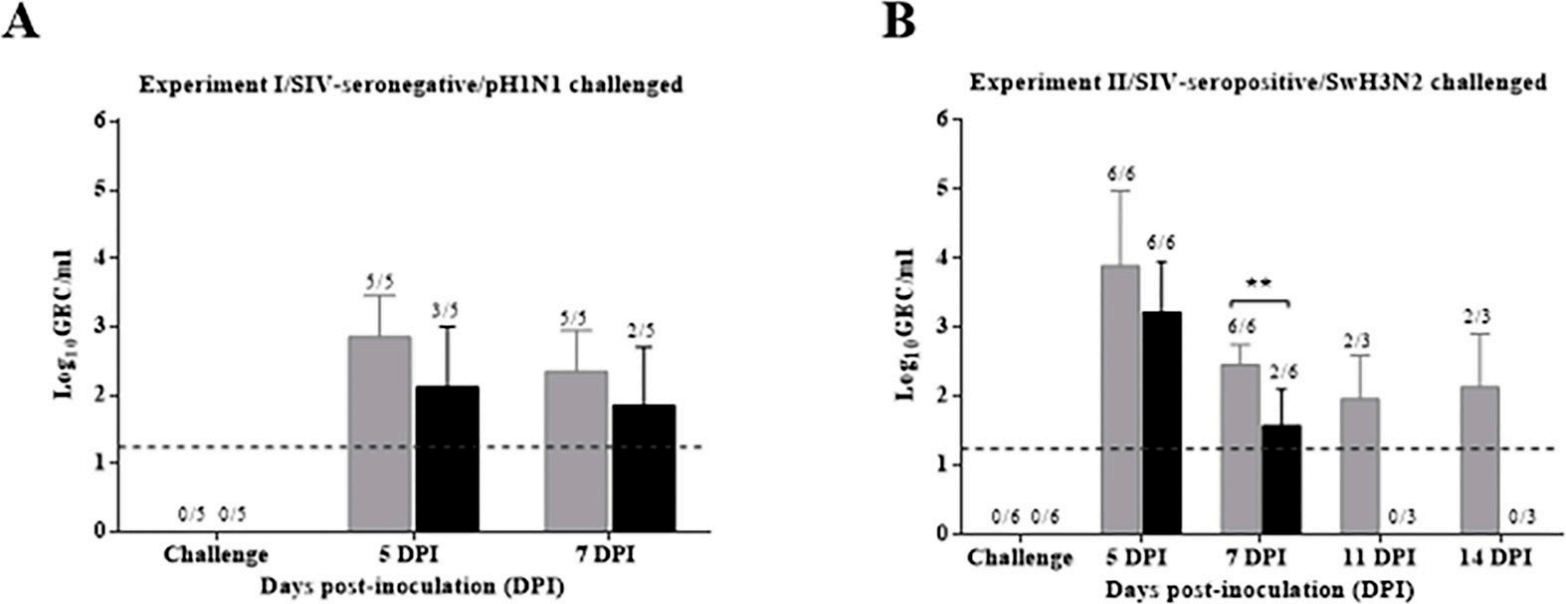
Viral RNA load in nasal swabs by RT-qPCR. (A) Mean of genomic equivalent copies (GEC) per mL obtained from nasal swabs from seronegative pigs (Experiment I) collected at 0, 5 and 7 dpi (B) and from nasal swabs from seropositive animals (Experiment II) collected at 0, 5, 7, 11 and 14 dpi. Group A (unvaccinated animals) is represented by grey bars and Group B (pCDNA3.1(+)-VC-4-flagellin vaccinated group) by black bars. Dpi, days post-inoculation. Dashed lines indicate the detection limit of the assays: 1.24 log10GEC/mL. Error bars indicate the mean ± SEM.

A reduced mean of genomic equivalent copies (GEC) per mL was observed at 5 and 7 dpi in the vaccinated group compared to the unvaccinated group. Furthermore, two out of five animals cleared the virus at 5 dpi and a total of three out of five animals at 7 dpi. All pigs from the unvaccinated group showed viral RNA until the end of the experiment (Fig 1A). Moreover, a summarizing table with individual results of viral clearance and clinical symptoms is attached (S1 Table).

#### Vaccination using VC4-flagellin induced higher IgG titers in sera against both the H1 and H3 subtypes

**Fig 2:**
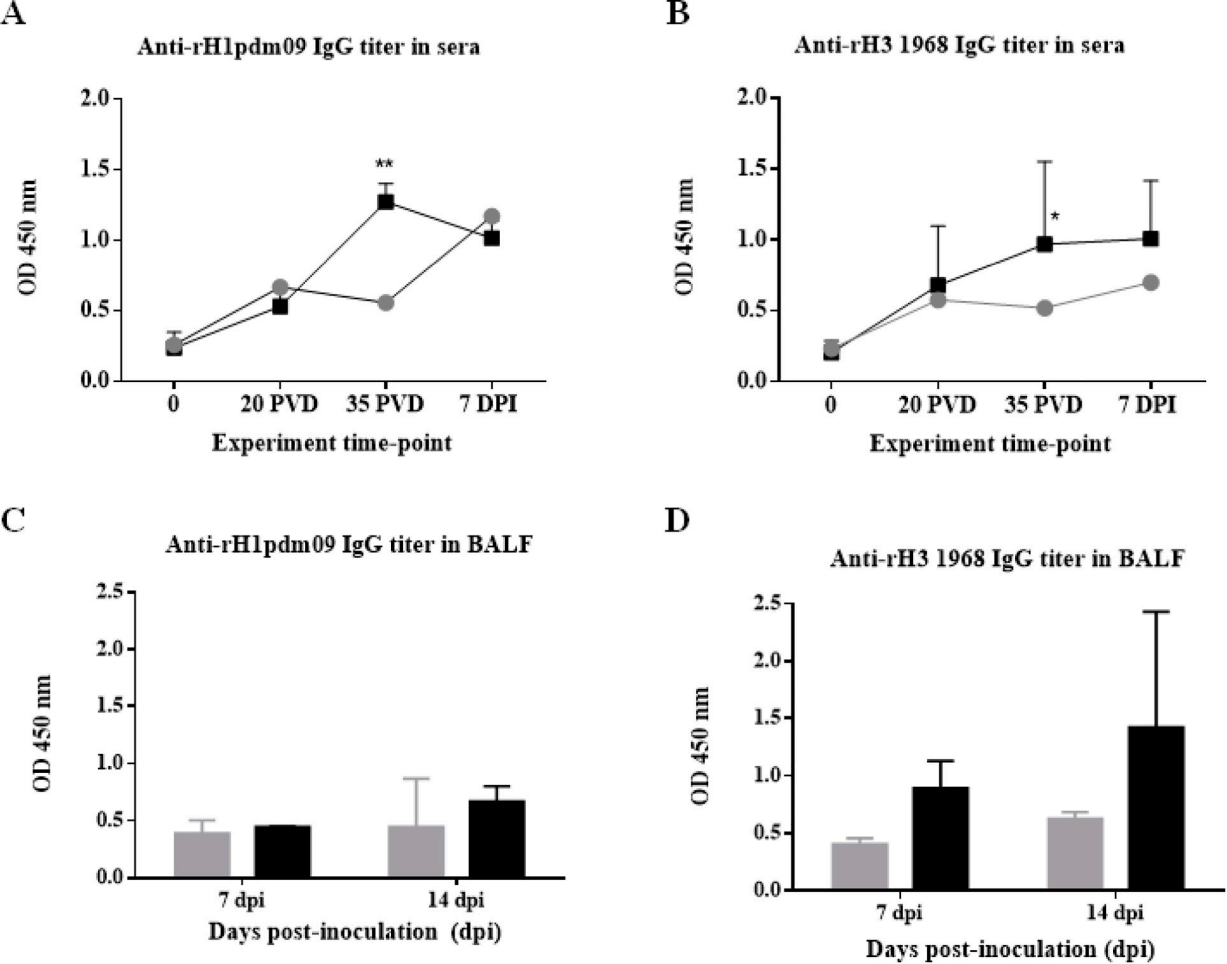
Serum antibody HA-specific IgG titers detected in sera and BALFs samples by ELISA test. Mean of serum IgG antibody levels detected at 0, 20 PVD, 35 PVD, and 7 DPI of Groups A and B (A) against HA from A/California/04/09(H1N1)pdm09, and (B) against HA from A/Aichi/2/1968(H3N2) are represented. Mean of BALFs IgG antibody levels detected in pigs sacrificed at 7 and 14 dpi of Groups A and B (C) against HA from A/California/04/09(H1N1)pdm09, and (D) against HA from A/Aichi/2/1968(H3N2). Grey circles/bars refer to group A (unvaccinated group), and black squares/bars refer to group B (pCDNA3.1(+)-VC4-flagellin vaccinated group). OD, optical density. PVD, post-vaccination days and DPI, days post-inoculation. Error bars indicate the mean ± SEM. Statistically significant differences between vaccine treatment groups (P value <0.05) are marked with *: *P*<0.05, **: *P*<0.01.

Pigs immunized with the VC4-flagellin vaccine manifested a boost in IgG antibodies against H1 and H3 subtypes in sera compared to unvaccinated groups, being the peak at 35 post-vaccination days (PVD) (before the challenge) (Fig 2A and 2B). The increased antibody level was significant (*P*<0.01) for H1 subtype. In comparison to the H3 subtype, the increment in the antibody levels at 35 PVD was also significant, but with a higher *p* value (*p*<0.05). This difference could probably be attributed to one particular animal in this group (pig 10: vaccinated challenged with pH1N1) that, unlike the four other animals from the VC4-flagellin group, did not show seroconversion against H3 neither upon vaccination with VC4-flagellin nor after challenge with pH1N1 influenza virus (IV).

#### Vaccination using VC-4 flagellin promoted higher HI titers in sera against pH1N1

**Fig 3:**
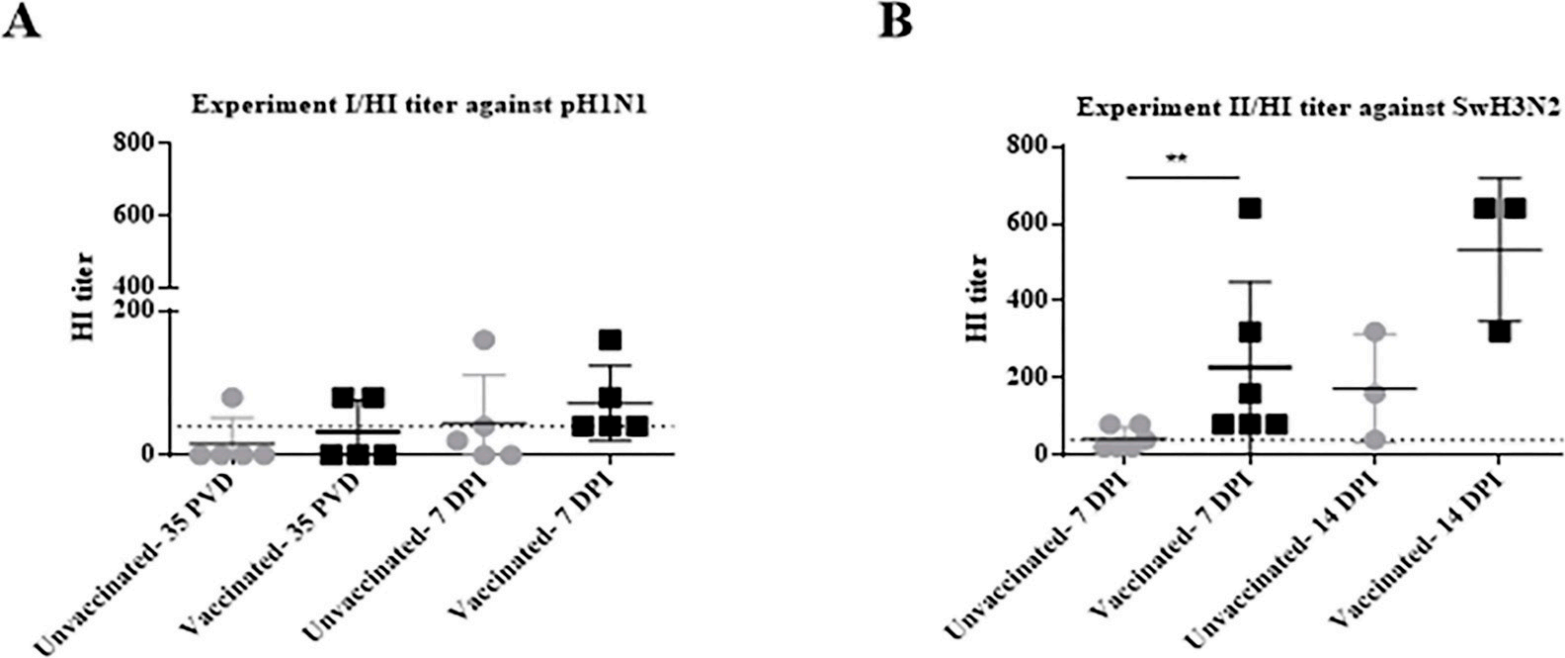
HI activity against pH1N1 from seronegative pigs (Experiment I) and against H3N2 from seropositive pigs (Experiment II). HI titers obtained with sera from unvaccinated (Group A) and vaccinated (Group B) pigs against (A) the pH1N1 from SIV-seronegative pigs (Experiment I) and (B) the SwH3N2 from SIV-seropositive pigs (Experiment II). Grey circles refer to group A (unvaccinated group) and black squares depict group B (pCDNA3.1(+)-VC-4-flagellin vaccinated group). HI, hemagglutination inhibition. DPI, days post-inoculation. Dashed lines indicate the threshold to obtain a “seroprotective” titer (HI≥40) which has been used as a criterion of immunogenicity in a vaccine. Error bars indicate the mean ± SEM and statistically significant differences between vaccine treatment groups are marked with **: *P*<0.01.

Likewise, to discriminate whether the antibodies obtained in sera could also block viral entry, we carried out an HI assay against the pH1N1. Two of the five VC4-flagellin vaccinated pigs showed values≥40 before challenge (35 PVD) and, unexpectedly, one pig from the unvaccinated group (Fig 3A). Nevertheless, the differences among groups were more illustrative at 7 dpi, when all pigs from the VC4-flagellin vaccinated displayed HI titers≥40. In contrast, only two unvaccinated pigs (animal 11 and 12) obtained seroprotective titers (animal 1, 1:40; animal 12; 1:160) at 7 dpi (Fig 3A).

### Experiment II (SIV-seropositive pigs/challenged with SwH3N2)

#### Clinical and pathological evaluation

Clinical examination revealed that four animals of each group (unvaccinated and VC4-flagellin vaccinated) exhibited fever. These animals corresponded to pig number 1 (fever at 6 and 7 dpi), 2(fever at the challenge day), 5 (fever at 3, 4 dpi) and 6 (fever from 3 to 7 dpi) from the unvaccinated group. From the VC4-flagellin group, the pigs with fever were the number 7 (fever at 4 dpi), 10 (fever at 3 and 7 dpi), 11 (fever at 2, 5 and 7 dpi) and 12 (fever from 2 to 4 dpi). Referring to clinical signs, one unvaccinated pig (pig 1) was coughing at 3 dpi. Three pigs vaccinated with VC4-flagellin were also coughing: pig number 7 and 8 (both coughing at 3 dpi) and pig 11 (at 4 dpi).

Using ImageJ ® analysis tools, the percentage of affected lung area of pigs were examined. Results revealed that two out of the three unvaccinated pigs (pig 1 and 5) had multifocal pulmonary cranio-ventral consolidation lesions: 4.11% and 2.93%, respectively, observed on the dorsal side of the lung. From the VC4-flagellin vaccinated group only one pig (pig 9) had dorsally (3.04%) and ventrally (2.61%) visible macroscopic lesions. Lungs collected at 14 dpi did not have lesions.

Intranasal inoculation with SwH3N2 caused a mild infection in all pigs and minor histopathological changes observable at necropsy. No differences in the severity of microscopic lung lesions of vaccinated and unvaccinated animals were recorded. Representatively, observations detected at 7 and 14 dpi from the second experiment are depicted in Table 4. Other pathological findings documented were one unvaccinated pig (animal 1) and two vaccinated pigs (animal 7 and 9) with suppurative bronchopneumonia. In addition, one immunized pig (animal 8) had fibrous pleuritis.

**Table 4:** Pathological microscopic score for all the animals from Experiment II based on BIP-compatible lesions. BIP was assessed by a semi-quantitative scoring (0–3, indicating lack of, mild, moderate or severe pneumonia lesions, respectively).

Group	Animal Id	Dpi	BIP scoring
**A**: Unvaccinated group	1	7	2
	2		2
	5		0.5
	3	14	1.5
	4		3
	6		0.5
**B**: pCDNA3.1(+)-VC4-flagellin vaccinated group	7	7	3
	9		2
	12		3
	8	14	0.5
	10		1
	11		2

Abbreviations: BIP = broncho-interstitial pneumonia; dpi = days post-inoculation; Id = identification.

#### Vaccination using VC4-flagellin limited or reduced SwH3N2 viral load

The mean of GEC from the VC4-flagellin vaccinated group was lower than the unvaccinated group at 5 and 7 dpi. Notably, four out of six VC4-flagellin vaccinated pigs cleared the virus at 7 dpi. Conversely, none of the unvaccinated group was able to clear the virus at 7 dpi (*P*<0.01) (Fig 1B).The unvaccinated-infected pigs continued shedding influenza virus up to 14 dpi (Fig 1B) whereas IV virus was not detected in VC4-flagellin vaccinated group (Fig 1B). Additionally, a summarizing table with individual results of viral clearance and clinical symptoms is attached (S2 Table).

#### Vaccination using VC4-flagellin induced superior IgG titers in BALFs against both the H1 and H3 subtypes

The presence of specific antibodies against H1 and H3 was also examined in the BALF samples from seropositive animals. The average of IgG antibody values at 7 and 14 dpi of the H1 and H3 subtypes were higher in the vaccinated group (Fig 2C and 2D). Considering that the challenged virus in Experiment II was from H3 subtype, enhanced antibody values were expected against H3 (Fig 2D).

#### Vaccination using VC4-flagellin promoted higher HI titers in sera against SwH3N2

No evident seroconversion effect in the seropositive pigs against H1 and H3 subtypes could be observed. However, animals did show HI activities in sera after challenge. HI results against the SwH3N2 evidenced that the VC4-flagellin vaccinated pigs had higher HI titers at 7 and 14 dpi than the unvaccinated animals. At 7 dpi, all pigs from the VC4-flagellin vaccinated group exhibited a positive HI titer (≥40) (*P*<0.01). Contrarily, only three out of six animals of the unvaccinated group remained with seroprotective titers ≥40. In addition, at 14 dpi, the inhibiting capacity of the three remaining VC4-flagellin vaccinated animals (animal 7; 1:640; animal 11; 1:320, animal 12; 1:640) was higher than the remaining three unvaccinated (animal 3; 1:40, animal 4; 1:320; animal 5; 1:160) (Fig 3B).

#### Vaccination using VC4-flagellin induced stronger IgA responses in BALF samples

**Fig 4:**
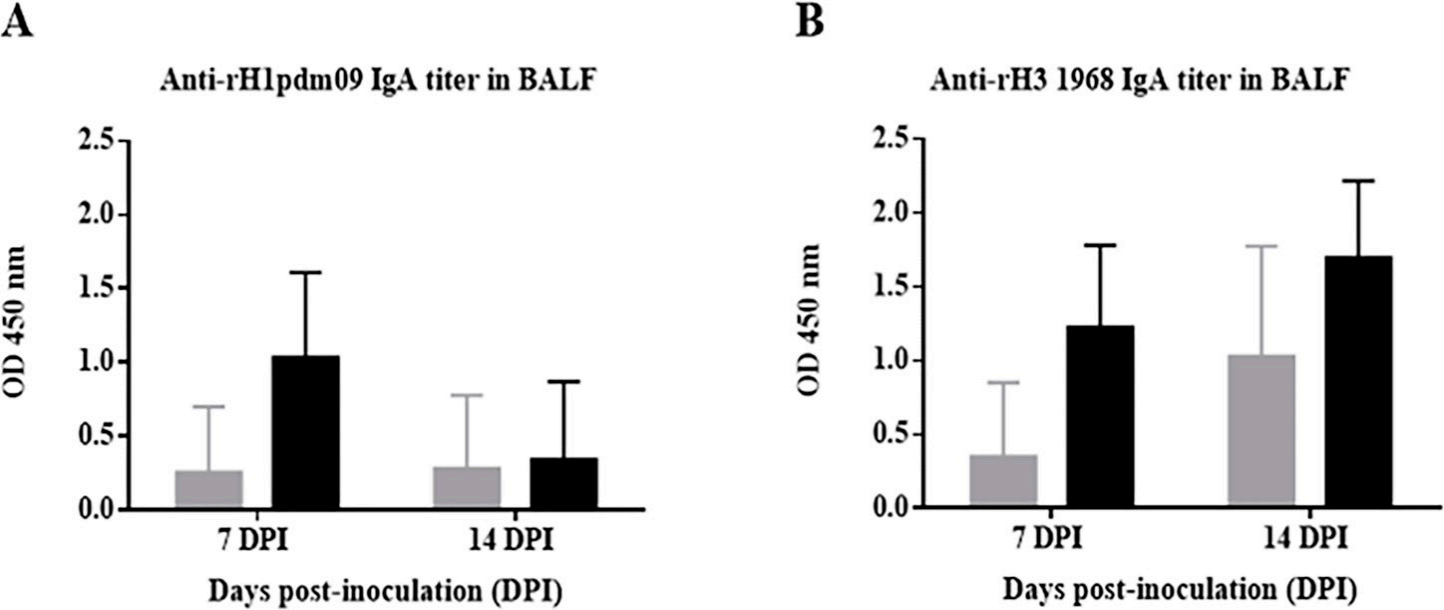
Antibody HA-specific IgA titers detected in BALFs samples from Experiment II. Mean of BALFs IgA antibody levels detected in pigs sacrificed at 7 and 14 dpi of Groups A and B (A) against HA from A/California/04/09(H1N1)pdm09, and (B) against HA from A/Aichi/2/1968(H3N2). Grey bars refer to group A (unvaccinated group), and black bars refer to group B (pCDNA3.1(+)-VC4-flagellin vaccinated group). OD, optical density. PVD, post-vaccination days and DPI, days post-inoculation. Error bars indicate the mean ± SEM.

The mucosal antibody response was investigated in the BALF samples. Against H1 subtype, vaccinated pigs elicited an increase in the IgA response in comparison to unvaccinated pigs at 7 dpi (Fig 4A). At 14 dpi, the same tendency was observed although the OD values of antibodies were less than at 7 dpi. At 7 and 14 dpi, the VC4-flagellin vaccinated pigs exhibited elevated IgA values compared to the unvaccinated pigs when analyzing IgAs against H3 subtype (Fig 4B).

#### Vaccination using VC4-flagellin promoted higher SNT titers in BALFs

**Fig 5:**
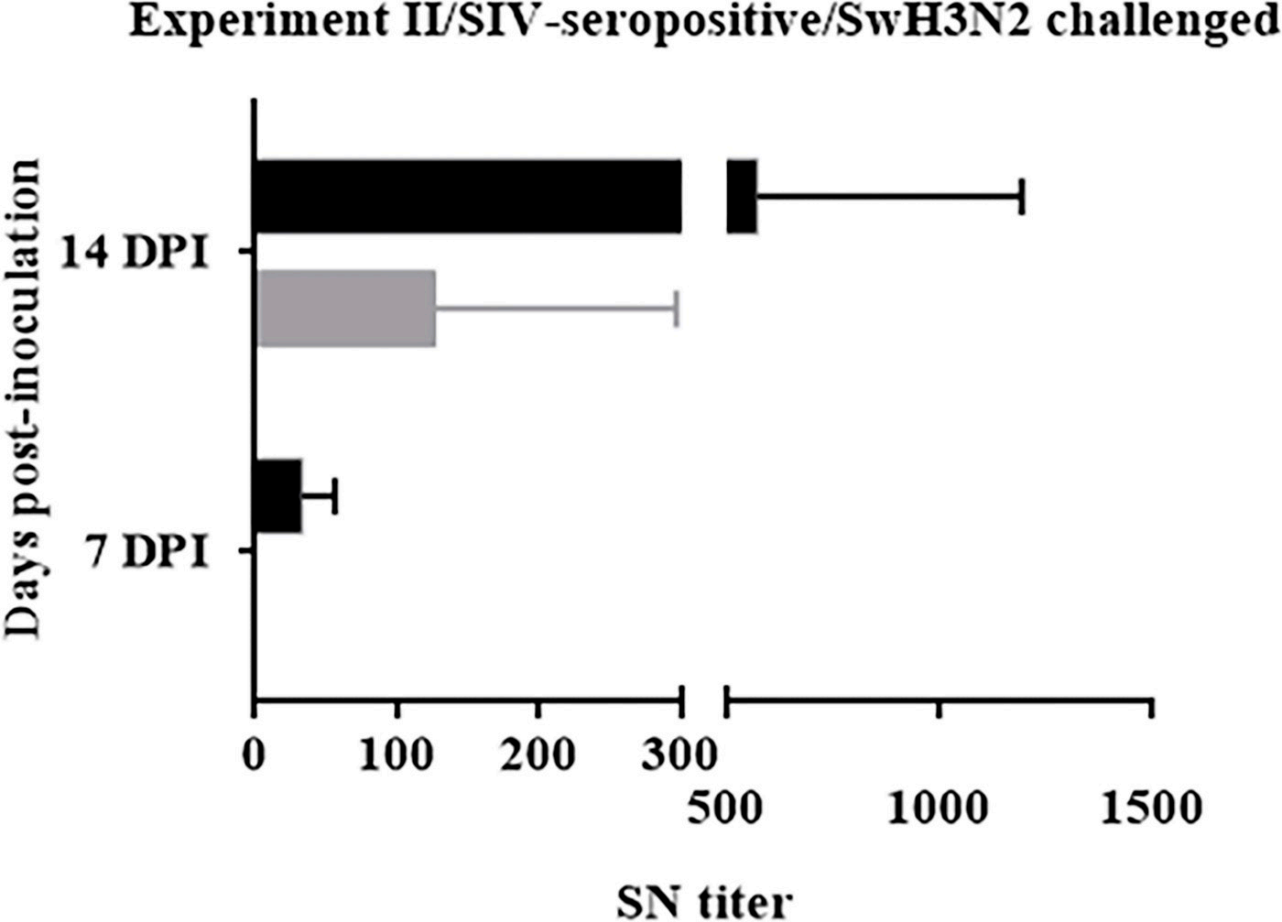
Seroneutralization (SN) titers detected in BALF samples from Experiment II by seroneutralization assay. Mean of seroneutralization titers detected at 7 and 14 dpi of Groups A and B against the A/swine/Spain/003/2010 H3N2 IV challenged virus. Grey bars refer to group A (unvaccinated group) and black bars depict group B (pCDNA3.1(+)-VC-4-flagellin vaccinated group). SN, seroneutralization. DPI, days post-inoculation. Error bars indicate the mean ± SEM.

After determining that the VC4-flagellin vaccinated group displayed higher antibody titers in the BALFs compared to the unvaccinated pigs, we were intrigued to find whether the elicited antibodies could neutralize the virus. VC4-flagellin vaccinated pigs showed higher mean values of seroneutralizing antibody titres in BALFs than the unvaccinated pigs at 7 and 14 dpi (Fig 5). Moreover, at 7 dpi, all animals from the vaccinated group manifested seroneutralizing titres (animal 6, value 1:20; animal 8, value 1:20; animal 9; 1:60). None of the unvaccinated animals developed seroneutralizing antibodies. At 14 dpi, two out of three unvaccinated pigs achieved seroneutralizing titres (animal 3, 1:60; animal 4, 1:320), but to lesser extent than immunized pigs (animal 8, value 1:320; animal 11, value 1:1280, animal 12, value 1:120).

#### Vaccination using VC4-flagellin promoted higher percentage of double-positive T-cells CD4-CD8

**Fig 6:**
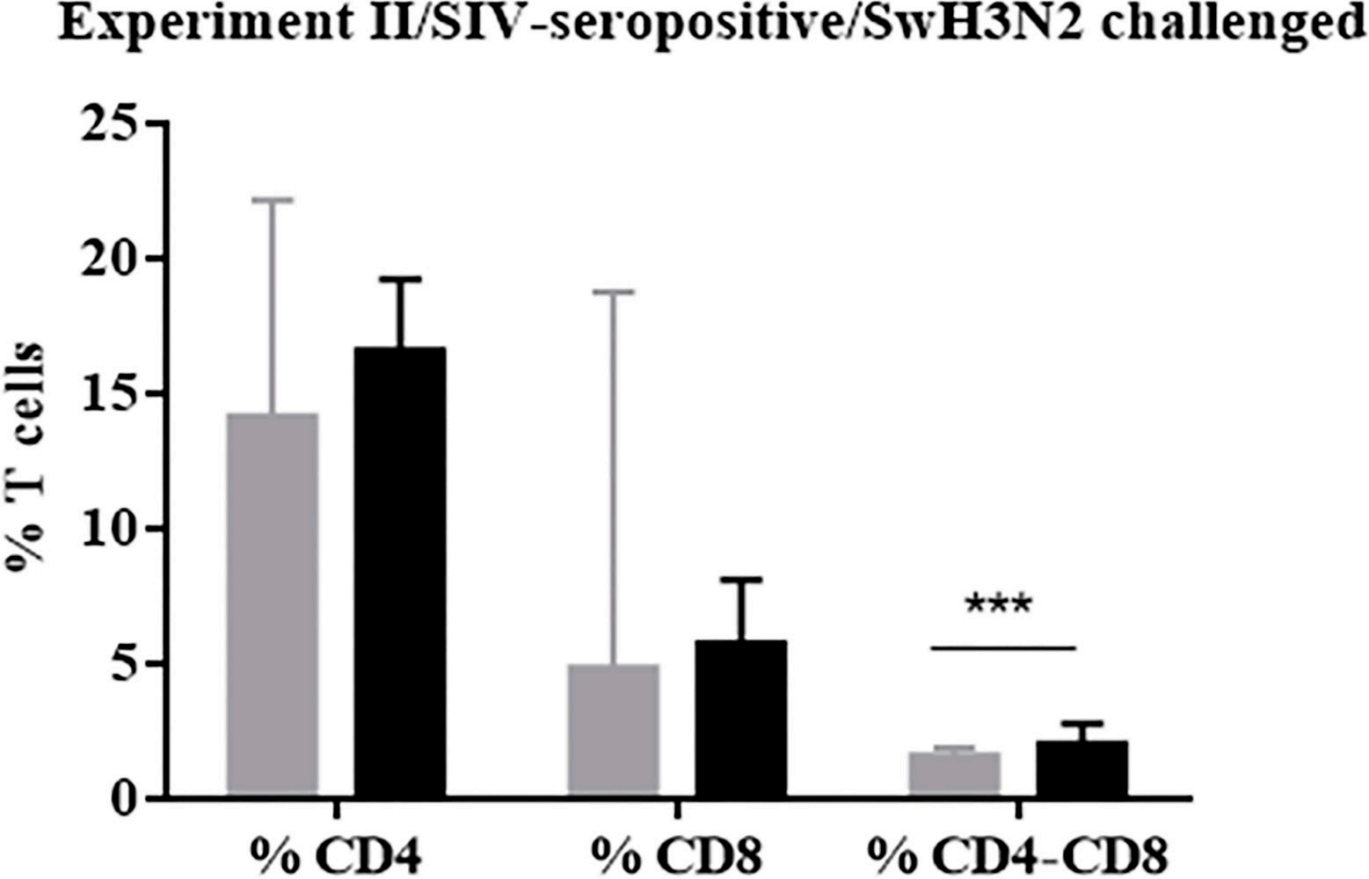
Flow cytometry from PBMCs isolated at 35 PVD (Experiment II). Mean of the percentages of T cells CD4, CD8 and CD4-CD8 DP from unvaccinated (Group A) and vaccinated (Group B). Grey bars refer to group A (unvaccinated group) and black bars depict group B (pCDNA3.1(+)-VC-4-flagellin vaccinated group). Error bars indicate the mean ± SEM and statistically significant differences between vaccine treatment groups are marked with ***: *P*<0.001.

A numeric increment of phenotypic population of T-cells CD4 SP (single positive) and T-cells CD8 SP was observed in the vaccinated group in comparison to the unvaccinated group prior to challenge; such increase was significant for CD4-CD8 DP (double positive) cells (*P*<0.001) (Fig 6).

## Discussion

Each year there is the demand to identify the strains of influenza A and B viruses that will be circulating in the next season, in order to manufacture the best option for seasonal influenza vaccines. Consequently, universal vaccines against influenza virus making use of highly conserved epitopes or proteins have been investigated during recent years. The present study describes how the combination of several conserved HA-peptides in a DNA approach constitutes a potential influenza vaccine for use in conventional pigs.

All HA-peptides included in the DNA approach presented were selected by means of the ISM platform, which is based on virtual spectroscopy [37,38]. HA-peptides were selected since well-matched antibodies to the HA can block an influenza virus infection and also contribute to the clearance of the virus from the lungs [51,52]. Furthermore, to obtain an improved presentation of the antigens and to enhance immunogenicity, flagellin was fused to the conserved HA-peptides. Such approach should result more potent and efficacious since incorporates TLR-ligands (such as flagellin) [53]. The flagellin ligand fused to an antigen of interest has been shown to yield vaccines able to induce higher IgG responses by means of improving antigen presenting cells (APCs) functions [53–57]. Moreover, flagellin induces TLR5 signaling and this pathway triggers the recruitment of granulocytes and macrophages/monocytes in the respiratory airways. Subsequently, the production of cytokines and chemokines required to initiate strong humoral and cellular responses is primed [39]. This characteristic is coherently related to the flagellin stimulation of monocytes to produce IL-10 and TNFα cytokines [58], of the NK cells to deliver IFNγ and α-defensins and of the T cells to proliferate and produce cytokines and chemokines (e.g. IL-10, IL-8 and IFNγ) [59]. Furthermore, it is a usual practice to include flagellin (FliC) as an adjuvant in novel universal vaccine approaches to face influenza viruses [53,60–64]. It is reported in those studies that conserved influenza epitopes linked to the flagellin either at the N or C terminus, or inclusive in its hypervariable region, did not impair the proper binding of the flagellin ligand to the TLR5.

Due to the final length of the construct and since DNA vaccines can provide the activation of both humoral and cellular responses, the construct VC4-flagellin was reverse-translated into a pCDNA3.1(+) plasmid. Moreover, DNA-based vaccines may cross-protect when facing heterologous swine influenza viruses without being as hazardous as the attenuated-typed [65]. Besides, a suitable delivery platform of the vaccine was sought. At the very end, an intradermal delivery approach seems to promote higher antibody titers than the intramusucular route [66–68]. The overall approach used was also selected because the optimal doses of DNA plasmid (moles) to be used were already described [69].

Thus, in this work, the VC-4-flagellin construct administered intradermally mixed with Diluvac Forte® adjuvant was tested as a vaccine candidate in pigs with or without MDA. Diluvac Forte® was mixed with the vaccine formulation but also was administered to unvaccinated pigs. Intriguingly, the VC4-flagellin vaccinated pigs demonstrated a reduction/clearance of the viral shedding in days 5 and 7 in Experiment I (seronegative animals, pH1N1 challenged) (Fig 1A) and in Experiment II (seropositive animals, H3N2 challenged) (Fig 1B). Therefore, we anticipate that MDA antibodies were not an apparent obstacle for the vaccine to reduce viral shedding and, eventually, to potentially block the viral transmission. Surprisingly, unlike unvaccinated pigs, seropositive vaccinated pigs did not shed the challenge H3N2 virus at 11 or 14 dpi although they were constantly in contact with unvaccinated infected animals (Fig 1B). This fact indicated that vaccination with VC4-flagellin not only limited the virus shedding from vaccinated pigs but, most possibly, also prevented re-infection in a contaminated environment. Further studies are needed to prove this assumption using contact infection experiments in larger groups.

Previous to challenge, the vaccinated animals of Experiment I could recognize both the H1 and H3 IV-subtypes (Fig 2A and 2B). In consequence, seroconversion and a cross-protecting effect against the two IV-subtypes were demonstrated. Apart from the post-vaccination seroconversion, the HI titers of ≥40 are considered to constitute a marker that correlates *in vitro* with protection [47–50]. Analyzing HI titers against pH1N1 (Experiment I, SIV-seronegative pigs/challenged with pH1N1) seroprotective antibodies could be found in 2 out of 5 pigs prior to the challenge. Noticeably, all the five vaccinated pigs manifested seroprotective titers at 7 dpi (Fig 3A). Moreover, seropositive vaccinated pigs elicited against SwH3N2 higher HI values at 7 and 14 dpi than the unvaccinated group, confirming that MDA were not interfering with the vaccine effect (Fig 3B).

Nevertheless, the vaccine failed to reduce the influenza clinical signs and lung lesions (Table 4). Indeed, no relevant differences were found between groups. Also, it is vital to take into consideration that the clinical picture and disease caused by the pH1N1 in pigs it is generally mild and subclinical [70,71]. In fact, in Experiment I very little number of pigs manifested fever or any clinical sign.

IgG and IgA antibody titers against H1 and H3 subtypes and their seroneutralizing effect against the challenge virus were analyzed in the BALFs of Experiment II. Overall, IgG antibodies were to a higher rate against both the H1 and H3 subtypes in DNA-vaccinated animal group than the unvaccinated group at 7 and 14 dpi (Fig 2C and 2D). The major difference among groups was observed at 14 dpi against the H3 subtype (Fig 2D). Assuming that the challenged virus in Experiment II was an H3N2 virus, elevated H3 antibodies were expected. Furthermore, a stronger seroneutralizing effect could be observed in the BALF samples obtained from the vaccinated pigs than in the samples from the unvaccinated ones (Fig 5). IgA antibodies were also to a higher rate against both the H1 and H3 subtypes in the vaccinated group than the unvaccinated one at 7 days pot-infection (Fig 4). A comparable tendency of IgA antibodies was observed in BALFs collected at 14 dpi against H3 subtype, indicating an enhanced mucosal immune response induced after VC-4-flagellin vaccination in pigs (Fig 4B). This is in line with studies claiming that mucosal immune response is necessary for the design of an universal influenza vaccine as it is the first line of defense against IVs [72,73]. We consider that mucosal immune response (IgA) elicited after VC-4-flagellin vaccination in pigs might have contributed in limiting virus shedding and cross-protection, as reported previously also by others [74,75].

VC-4-flagellin vaccination in pigs, interestingly, also showed an increase in the frequency of the CD4-CD8 DP T cells subset (Fig 6). In fact, results from an earlier report [76] evidenced that some CD4-CD8 DP T cell subset likely belong to effector memory T cells (T_EM_). This data was only analyzed at pre-challenge time point and not followed after the challenge. Further investigation would be necessary to ultimately define the role of the CD4-CD8 DP T cell subset in protection and clearance of IV after VC-4-flagellin vaccination in pigs.

Our results strongly indicate that HA specific immune response effectively contributed to control influenza infections after VC-4 flagellin vaccination without MDA apparent interference. Promoting a solid systemic mucosal response and blocking viral transmission by reducing earlier the viral shedding were the key outcomes in the VC4-flagellin vaccination approach. Therefore, VC4-flagellin as such maybe an interesting vaccine candidate against H1 and H3 subtypes. However, more studies are crucial in order to vaccinate with VC4-flagellin and mitigate clinical manifestations and lung pathology.

## Supporting information

S1 Checklist ARRIVE guidelines checklist fulfilled according to the parameters followed in both of the two swine experimental designs. (PDF)

S1 Fig Plots for gating CD4, CD8 SP T-lymphocytes and CD4-CD8 DP lymphocytes in flow cytometry. A) lymphocytes B) CD4, CD8 T-lymphocytes and CD4-CD8 DP lymphocytes plot C) CD4 T-lymphocytes, D) CD8 T-lymphocytes
(TIF)

S1 Table Individual animal GEC per mL of the nasal swabs samples collected from the 1^st^ experiment at 5 and 7 dpi. Clinical signs recorded for each of the animals are also depicted.
(PDF)

S2 Table Individual animal GEC per mL of the nasal swabs samples collected from the 2^nd^ experiment at 5, 7, 11 and 14 dpi. Clinical signs recorded for each of the animals are also depicted.
(PDF)

S3 Table Mean and mean of the standard deviation of the GEC per mL of the nasal swabs samples collected from the 1^st^ experiment at 0, 5 and 7. (PDF)

S4 Table Mean and mean of the standard deviation of the GEC per mL of the nasal swabs samples collected from the 2^nd^ experiment at 0, 5, 7, 11 and 14. (PDF)

S5 Table Mean and standard deviations of OD 450 nm IgG values obtained against HA of A/California/04/09(H1N1)pdm09 from sera samples for each triplicate at 0, 20PVD, 35PVD and 7 dpi. (PDF)

S6 Table Mean and standard deviation of OD 450 nm IgG values obtained against HA from A/Aichi/2/1968(H3N2) from sera samples for each triplicate at 0, 20PVD, 35PVD and 7 dpi. (PDF)

S7 Table Mean and standard deviation of OD 450 nm IgG values obtained against HA from A/California/04/09(H1N1)pdm09 from BALFs samples for each triplicate at 7 and 14 dpi. (PDF)

S8 Table Mean and standard deviation of OD 450 nm IgG values obtained against HA from A/Aichi/2/1968(H3N2) from BALFs samples for each triplicate at 7 and 14 dpi. (PDF)

S9 Table Individual animal mean HI titer obtained against virus A/Catalonia/63/2009 H1N1 IV from sera samples for each duplicate at 35 PVD and 7 dpi (1^st^ experiment). (PDF)

S10 Table Individual animal mean HI titer obtained against virus A/swine/Spain/003/2010 H3N2 IV from sera samples for each duplicate at 7 dpi and 14 dpi (2^nd^ experiment). (PDF)

S11 Table IgA Mean and standard deviation of OD 450 nm IgA values obtained against HA from A/California/04/09(H1N1)pdm09 from BALF samples for each triplicate at 7 dpi and 14 dpi. (PDF)

S12 Table Mean and standard deviation of OD 450 nm IgA values obtained against HA from A/Aichi/2/1968(H3N2) from BALF samples for each triplicate at 7 and 14 dpi. (PDF)

S13 Table Mean and standard deviation of SNT titers obtained against A/swine/Spain/003/2010 H3N2 IV from sera samples for each duplicate at 7 and 14 dpi. (PDF)

S14 Table Mean and standard deviations of % of CD4, CD8 and CD4-CD8 T cells from PBMCs samples for each duplicate at prechallenge time-point (2^nd^ experiment). (PDF)
