# Supplementary figures and images for: DNA vaccine based on conserved HA-peptides induces strong immune response and rapidly clears influenza virus infection from vaccinated pigs
Source: PLoS One. 2019 Sep 25;14(9):e0222201. doi: 10.1371/journal.pone.0222201 (PMC6760788; doi:10.1371/journal.pone.0222201)

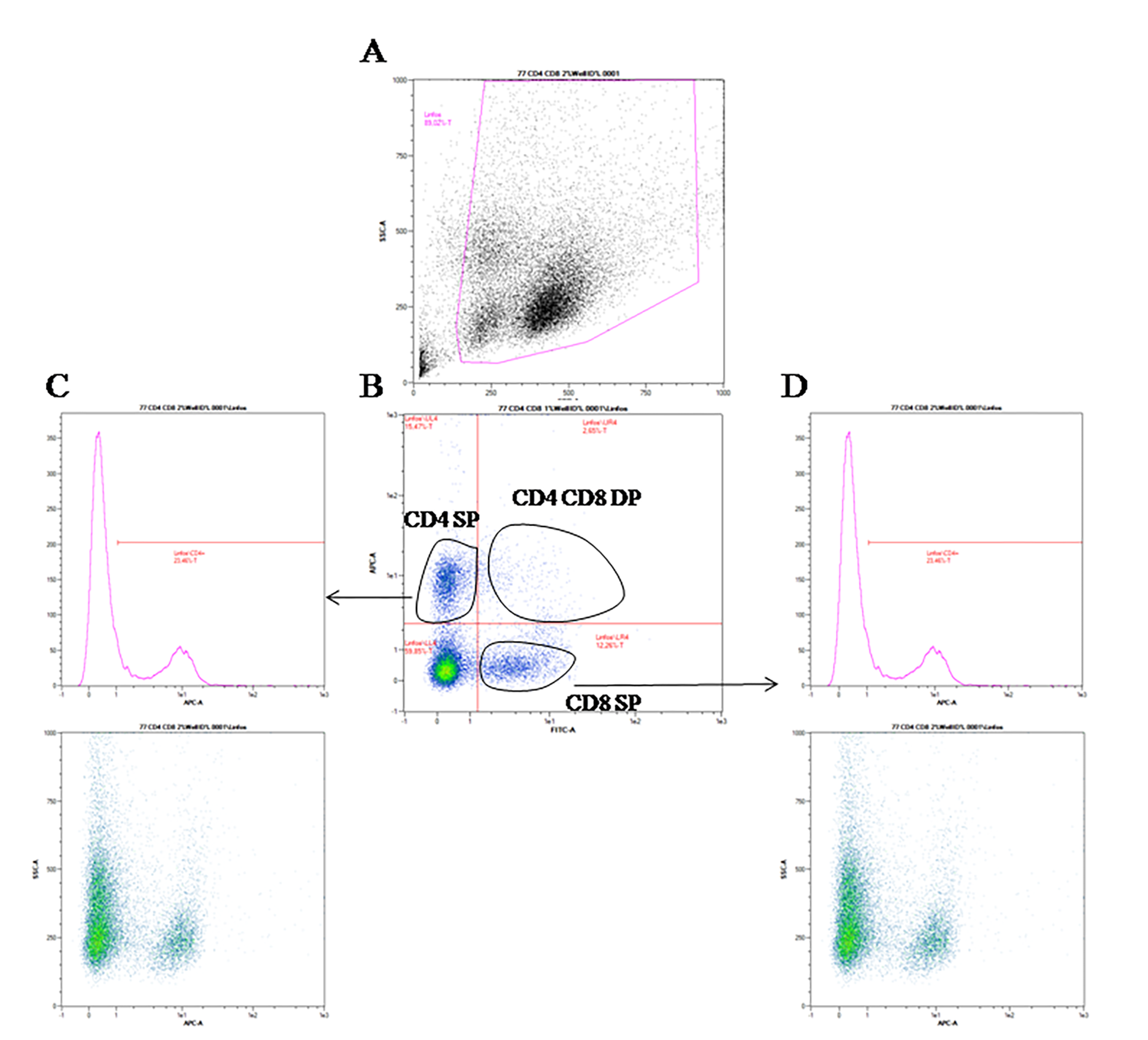

Supplement: S1 Fig — A) lymphocytes B) CD4, CD8 T-lymphocytes and CD4-CD8 DP lymphocytes plot C) CD4 T-lymphocytes, D) CD8 T-lymphocytes (TIF) [file pone.0222201.s002.tif]
