# Supplementary material for: DNA vaccine based on conserved HA-peptides induces strong immune response and rapidly clears influenza virus infection from vaccinated pigs
Source: PLoS One. 2019 Sep 25;14(9):e0222201. doi: 10.1371/journal.pone.0222201 (PMC6760788; doi:10.1371/journal.pone.0222201)
Supplement: S1 Table — Clinical signs recorded for each of the animals are also depicted. (PDF) [file pone.0222201.s003.pdf]

S1 Table. Individual animal GEC per mL of the nasal swabs samples collected from the 1st experiment at 5 and 7 dpi. Clinical signs recorded for each of the animals are also depicted.

**Group**	**Animal Id**	**Log $_{10}$ GEC/mL**		**Clinical signs**
		**5 dpi**	**7 dpi**	
Unvaccinated	1	3,999	2,175	Fever 6 dpi
	2	2,349	2,750	Loose feces 7 dpi
	3	2,370	3,186	
	4	2,620	2,571	
	5	2,914	2,818	
Vaccinated	6	2,964	3,090	
	7	**1,240**	**1,240**	
	8	3,050	**1,240**	Fever 2 dpi
	9	**1,240**	2,420	
	10	2,098	**1,240**	

Numbers in bold type represent samples from which viral RNA was not detected (there were given the value of the detection limit of the technique).
