# Supplementary material for: DNA vaccine based on conserved HA-peptides induces strong immune response and rapidly clears influenza virus infection from vaccinated pigs
Source: PLoS One. 2019 Sep 25;14(9):e0222201. doi: 10.1371/journal.pone.0222201 (PMC6760788; doi:10.1371/journal.pone.0222201)
Supplement: S2 Table — Clinical signs recorded for each of the animals are also depicted. (PDF) [file pone.0222201.s004.pdf]

IRTA RESEARCH & TECHNOLOGY FOOD & AGRICULTURE
CReSA building
UAB Campus
08193 Bellaterra
Tel. +34 902 78 94 49
www.irta.cat
www.cresa.es
CReSA Centre de Recerca en Sanitat Animal

S2 Table. Individual animal GEC per mL of the nasal swabs samples collected from the 2nd experiment at 5, 7, 11 and 14 dpi. Clinical signs recorded for each of the animals are also depicted.

Group	Animal Id	Log $_{10}$ GEC/mL				Clinical signs
		5 dpi	7 dpi	11 dpi	14 dpi	
Unvaccinated	1	2,404	2,488			Fever at 6-7 dpi /coughing at 3 dpi
	2	3,560	2,169			Fever at day of challenge
	3	5,575	2,441	**1,240**	**1,240**	
	4	4,425	2,654	2,370	2,627	
	5	4,125	2,098			Fever 3,4 dpi
	6	3,232	2,880	2,277	2,528	Fever 3-7 dpi
Vaccinated	7	3,631	2,336			Fever (4 dpi)/ coughing at 3 dpi
	8	4,329	**1,240**	**1,240**	**1,240**	Coughing at 3 dpi
	9	2,933	2,163			
	10	3,473	**1,240**	**1,240**	**1,240**	Fever at 3 and 7 dpi
	11	2,413	**1,240**	**1,240**	**1,240**	Fever at 2, 5 and 7 dpi/ coughing at 4 dpi
	12	2,519	**1,240**			Fever at 2 to 4 dpi

Numbers in bold type represent samples from which viral RNA was not detected (there were given the value of the detection limit of the technique).
