# Supplementary material for: DNA vaccine based on conserved HA-peptides induces strong immune response and rapidly clears influenza virus infection from vaccinated pigs
Source: PLoS One. 2019 Sep 25;14(9):e0222201. doi: 10.1371/journal.pone.0222201 (PMC6760788; doi:10.1371/journal.pone.0222201)
Supplement: S3 Table — (PDF) [file pone.0222201.s005.pdf]

S3 Table. Mean and mean of the standard deviation of the GEC per mL of the nasal swabs samples collected from the 1st experiment at 0, 5 and 7.

	Viral shedding in nasal swabs (1st experiment)			
	Group A- Unvaccinated group		Group B- VC4-flagellin vaccinated	
Time-point	Mean Log_{10}GEC/mL	Mean SD	Mean Log_{10}GEC/mL	Mean SD
0	Negative	Negative	Negative	Negative
5	2,85	0,61	2,11	0,88
7	2,70	0,37	1,84	0,86
