# Supplementary material for: DNA vaccine based on conserved HA-peptides induces strong immune response and rapidly clears influenza virus infection from vaccinated pigs
Source: PLoS One. 2019 Sep 25;14(9):e0222201. doi: 10.1371/journal.pone.0222201 (PMC6760788; doi:10.1371/journal.pone.0222201)
Supplement: S4 Table — (PDF) [file pone.0222201.s006.pdf]

S4 Table. Mean and mean of the standard deviation of the GEC per mL of the nasal swabs samples collected from the 2nd experiment at 0, 5, 7, 11 and 14.

	**Viral shedding in nasal swabs (2nd study)**			
	**Group A- Unvaccinated group**		**Group B- VC4-flagellin vaccinated**	
**Time-point**	**Mean Log_{10}GEC/mL**	**Mean SD**	**Mean Log_{10}GEC/mL**	**Mean SD**
0	Negative	Negative	Negative	Negative
5	3,88	1,089	3,21	0,733
7	2,45	0,29	1,57	0,524
11	1,96	0,63	0	0
14	2,13	0,77	0	0
