# Supplementary material for: DNA vaccine based on conserved HA-peptides induces strong immune response and rapidly clears influenza virus infection from vaccinated pigs
Source: PLoS One. 2019 Sep 25;14(9):e0222201. doi: 10.1371/journal.pone.0222201 (PMC6760788; doi:10.1371/journal.pone.0222201)
Supplement: S5 Table — (PDF) [file pone.0222201.s007.pdf]

S5 Table. Mean and standard deviations of OD 450 nm IgG values obtained against HA of A/California/04/09(H1N1)pdm09 from sera samples for each triplicate at 0, 20PVD, 35PVD and 7 dpi.

	**Anti-rH1pdm09 OD 450nm IgG values in sera (1st experiment)**			
	**Group A- Unvaccinated group**		**Group B- VC4-flagellin vaccinated**	
**Time-point**	**Mean**	**SD**	**Mean**	**SD**
0	0,259	0,117	0,232	0,111
20 PVD	0,667	0,284	0,531	0,140
35 PVD	0,558	0,344	1,274	0,128
7 DPI	1,173	0,105	1,017	0,126
