# Supplementary material for: DNA vaccine based on conserved HA-peptides induces strong immune response and rapidly clears influenza virus infection from vaccinated pigs
Source: PLoS One. 2019 Sep 25;14(9):e0222201. doi: 10.1371/journal.pone.0222201 (PMC6760788; doi:10.1371/journal.pone.0222201)
Supplement: S6 Table — (PDF) [file pone.0222201.s008.pdf]

S6 Table. Mean and standard deviation of OD 450 nm IgG values obtained against HA from A/Aichi/2/1968(H3N2) from sera samples for each triplicate at 0, 20PVD, 35PVD and 7 dpi.

	Anti-rH3 1968 OD 450nm IgG values in sera (1st experiment)			
	Group A- Unvaccinated group		Group B- VC4-flagellin vaccinated	
Time-point	Mean	SD	Mean	SD
0	0,232	0,116	0,206	0,081
20 PVD	0,576	0,239	0,678	0,421
35 PVD	0,520	0,068	0,971	0,582
7 DPI	0,699	0,064	1,010	0,407
