# Supplementary material for: DNA vaccine based on conserved HA-peptides induces strong immune response and rapidly clears influenza virus infection from vaccinated pigs
Source: PLoS One. 2019 Sep 25;14(9):e0222201. doi: 10.1371/journal.pone.0222201 (PMC6760788; doi:10.1371/journal.pone.0222201)
Supplement: S9 Table — (PDF) [file pone.0222201.s011.pdf]

S9 Table. Individual animal mean HI titer obtained against virus A/Catalonia/63/2009 H1N1 IV from sera samples for each duplicate at 35 PVD and 7 dpi (1st experiment).

HI titer against pH1N1 in sera (1st experiment)					
**Animal**	**Group A-Unvaccinated group (35 PVD)**	**Group A-Unvaccinated group (7 DPI)**	**Animal**	**Group B-VC4-flagellin vaccinated (35 PVD)**	**Group B-VC4-flagellin vaccinated (7 DPI)**
1	0	40	6	80	160
2	80	160	7	0	40
3	0	0	8	0	40
4	0	0	9	0	40
5	0	20	10	80	80
