# Supplementary material for: DNA vaccine based on conserved HA-peptides induces strong immune response and rapidly clears influenza virus infection from vaccinated pigs
Source: PLoS One. 2019 Sep 25;14(9):e0222201. doi: 10.1371/journal.pone.0222201 (PMC6760788; doi:10.1371/journal.pone.0222201)
Supplement: S10 Table — (PDF) [file pone.0222201.s012.pdf]

S10 Table. Individual animal mean HI titer obtained against virus A/swine/Spain/003/2010 H3N2 IV from sera samples for each duplicate at 7 dpi and 14 dpi (2nd experiment).

HI titer against SwH3N2 in sera (2nd experiment)					
**Animal**	**Group A-Unvaccinated group (7 DPI)**	**Group A-Unvaccinated group (14 DPI)**	**Animal**	**Group B-VC4-flagellin vaccinated (7 DPI)**	**Group B-VC4-flagellin vaccinated (14 DPI)**
1	80		7	80	
2	20		8	320	640
3	20	40	9	640	
4	80	320	10	80	
5	20		11	80	320
6	40	160	12	160	640
