# Supplementary material for: DNA vaccine based on conserved HA-peptides induces strong immune response and rapidly clears influenza virus infection from vaccinated pigs
Source: PLoS One. 2019 Sep 25;14(9):e0222201. doi: 10.1371/journal.pone.0222201 (PMC6760788; doi:10.1371/journal.pone.0222201)
Supplement: S11 Table — (PDF) [file pone.0222201.s013.pdf]

**S11 Table. Mean and standard deviation of SNT titers obtained against A/swine/Spain/003/2010 H3N2 IV from sera samples for each duplicate at 7 and 14 dpi.**

| | Against SwH3N2 SNT titer in sera (2nd study) | | | |
|---|---|---|---|---|
| | Group A- Unvaccinated group | | Group B- VC4-flagellin vaccinated | |
| **Time-point** | **Mean SNT titer** | **Mean SD** | **Mean SNT titer** | **Mean SD** |
| 7 DPI | 0 | 0 | 33,3 | 23,09 |
| 14 DPI | 126,66 | 170,1 | 573,33 | 620,11 |
