# Supplementary material for: DNA vaccine based on conserved HA-peptides induces strong immune response and rapidly clears influenza virus infection from vaccinated pigs
Source: PLoS One. 2019 Sep 25;14(9):e0222201. doi: 10.1371/journal.pone.0222201 (PMC6760788; doi:10.1371/journal.pone.0222201)
Supplement: S12 Table — (PDF) [file pone.0222201.s014.pdf]

S12 Table. IgA Mean and standard deviation of OD 450 nm IgA values obtained against HA from A/California/04/09(H1N1)pdm09 from BALF samples for each triplicate at 7 dpi and 14 dpi.

	Anti-rH1pdm09 OD 450nm IgA values in BALFs (2nd experiment)			
	Group A- Unvaccinated group		Group B- VC4-flagellin vaccinated	
**Time-point**	**Mean**	**SD**	**Mean**	**SD**
7 DPI	0,256	0,444	1,093	0,573
14 DPI	0,285	0,494	0,345	0,525
