# Supplementary material for: DNA vaccine based on conserved HA-peptides induces strong immune response and rapidly clears influenza virus infection from vaccinated pigs
Source: PLoS One. 2019 Sep 25;14(9):e0222201. doi: 10.1371/journal.pone.0222201 (PMC6760788; doi:10.1371/journal.pone.0222201)
Supplement: S13 Table — (PDF) [file pone.0222201.s015.pdf]

S13 Table. Mean and standard deviation of OD 450 nm IgA values obtained against HA from A/Aichi/2/1968(H3N2) from BALF samples for each triplicate at 7 and 14 dpi.

	**Anti-rH3 1968 OD 450nm IgA values in BALFs (1st experiment)**			
	**Group A- Unvaccinated group**		**Group B- VC4-flagellin vaccinated**	
**Time-point**	**Mean**	**SD**	**Mean**	**SD**
7 DPI	0,351	0,502	1,231	0,552
14 DPI	1,031	0,745	1,698	0,520
