# Supplementary material for: DNA vaccine based on conserved HA-peptides induces strong immune response and rapidly clears influenza virus infection from vaccinated pigs
Source: PLoS One. 2019 Sep 25;14(9):e0222201. doi: 10.1371/journal.pone.0222201 (PMC6760788; doi:10.1371/journal.pone.0222201)
Supplement: S14 Table — (PDF) [file pone.0222201.s016.pdf]

S14 Table. Mean and standard deviations of % of CD4, CD8 and CD4-CD8 T cells from PBMCs samples for each duplicate at prechallenge time-point (2nd experiment).

	% of T-cells at pre-challenge time point (2nd experiment)			
	Group A- Unvaccinated group		Group B- VC4-flagellin vaccinated	
**T cells**	**Mean**	**SD**	**Mean**	**SD**
CD4 SP	14,22	7,98	16,82	2,62
CD8 SP	4,90	13,91	6,50	2,34
CD4-CD8 DP	1,63	0,2912	3,37	0,74
